# Acceptance and attitude towards the traditional chinese medicine among asymptomatic COVID-19 patients in Shanghai Fangcang hospital

**DOI:** 10.1186/s12906-023-03922-z

**Published:** 2023-03-30

**Authors:** Bo Pan, Hong-wei Yin, Yue Yu, Xing Xiang, Cui Yu, Xiao-Jie Yan, Xiao-feng Zhai, Yuan Bai, Jing Hong

**Affiliations:** 1 The First Affiliated Hospital of Naval Medical University, Shanghai, 200433 China; 2 School of Traditional Chinese Medicine, Naval Medical University, Shanghai, 200433 China; 3 The First Clinical Medical College, Nanjing University of Traditional Chinese Medicine, Nanjing, Jiangsu Province 210023 China; 4 Yueyang Hospital of Integrated Traditional Chinese and Western Medicine, Shanghai University of Traditional Chinese Medicine, Shanghai, 200437 China

**Keywords:** COVID-19, Traditional chinese medicine, Asymptomatic COVID-19 patients, Predictors, Cross-sectional study

## Abstract

**Objective:**

The Coronavirus Disease 2019 (COVID-19) has brought severe damage to global health and socioeconomics. In China, traditional Chinese medicine (TCM) is the most important complementary and alternative medicine (CAM) and it has shown a beneficial role in the prevention and treatment of COVID-19. However, it is unknown whether patients are willing to accept TCM treatment. The objective of our study is to investigate the acceptance, attitude, and independent predictors of TCM among asymptomatic COVID-19 patients admitted to Shanghai fangcang hospital during the outbreak of the COVID-19 pandemic in Shanghai in 2022.

**Methods:**

A cross-sectional study was conducted on asymptomatic COVID-19 patients in the largest fangcang hospital in Shanghai, China, from April 22, 2022, to May 25, 2022. Based on the literature review of previous similar studies, a self-report questionnaire was developed to assess the patients’ attitude and acceptance of TCM, and a multivariate logistic regression analysis was conducted to determine the independent predictors of TCM acceptance.

**Results:**

A total of 1,121 patients completed the survey, of whom 91.35% were willing to accept CAM treatment whereas 8.65% of participants showed no willingness. Multivariate logistic regression analysis revealed that the patients who have received two doses of COVID-19 vaccine (OR = 2.069, 95%CI: 1.029–4.162, *P* = 0.041 vs. not received), understood the culture of TCM (OR = 2.293, 95%CI: 1.029–4.162, *P* = 0.014 vs. not understood), thought the TCM treatment is safe (OR = 2.856, 95%CI: 1.334–6.112, *P* = 0.007 vs. not thought), thought the TCM treatment is effective (OR = 2.724, 95%CI: 1.249–5.940, *P* = 0.012 vs. not thought), and those who informed their attending physician if using TCM for treatment (OR = 3.455, 95%CI:1.867–6.392, *P* < 0.001 vs. not informed) were more likely to accept TCM treatment. However, patients who thought TCM might delay your treatment (OR = 0.256, 95%CI: 0.142–0.462, *P* < 0.001 not thought) was an independent predictor for unwillingness to accept TCM treatment.

**Conclusion:**

This study preliminarily investigated the acceptance, attitude, and predictors of intention to receive TCM among asymptomatic COVID-19 patients. It is recommended to increase the publicity of TCM, clarify the impact of TCM and communicate with attending doctors that meet the healthcare needs of asymptomatic COVID-19 patients.

## Background

At present, the Coronavirus Disease 2019 (COVID-19) is still spreading all over the world, which has brought a heavy burden to the global economic recovery and made the fragile healthcare system of some developing countries fall into a state of being on the verge of collapse. The daily number of confirmed cases presents a rising trajectory across the globe. As of January 27, 2023, the total number of confirmed cases had reached over 752 million with more than 6.80 million deaths worldwide [1].

Complementary and alternative medicine (CAM) is defined as a set of different medical and healthcare systems, practices, and products, which are generally not considered part of traditional medicine [2]. One survey carried out in Iran between April 20, 2020, and August 20, 2020, demonstrated that at least one type of CAM was used by 84% of the participants during the outbreak of the COVID-19 pandemic. In the majority of participants, CAM was adopted to prevent the infection of COVID-19 or reduce the anxiety caused by the COVID-19 pandemic [3]. An anonymous electronic survey was conducted in Ghana showed that 82.5% of the participants applied CAM during COVID-19 [4]. A meta-analysis showed that different CAM interventions such as acupuncture, traditional Chinese medicine (TCM), relaxation, and Gongfa significantly alleviated the psychological symptoms (depression, anxiety, stress, sleep quality, negative emotions, quality of life) and physical symptoms (inflammatory factors, physical activity, chest pain, and respiratory function) in COVID-19 patients [5].

As an important part of CAM in China, TCM has its characteristics in the prevention and treatment of acute infectious diseases [6, 7], which is one of the major therapies recommended in the guideline for prevention, control, diagnosis, and treatment of COVID-19 published by the National Health Commission of China [8]. A clinical study conducted at Wuhan fangcang hospitals found that early treatment with Huashibaidu granule for seven days alleviated the deterioration of symptoms in COVID-19 patients with moderate and mild symptoms [9]. Another study also showed that Lianhua Qingwen capsules could significantly shorten the median time of recovery and improve the improvement rate of chest CT imaging as well as the clinical cure rate. Moreover, the role of TCM in the prevention and control of COVID-19 has been recognized by the World Health Organization [10].

In the last week of February 2022, the Omicron BA 2.2 variant of COVID-19 caused a wave of infection in Shanghai, China [11]. Asymptomatic COVID-19 patients were sent to Fangcang hospitals by the community, which were upgraded to designated hospitals for the treatment of asymptomatic COVID-19 patients. To apply TCM to more asymptomatic COVID-19 patients, it is particularly important to understand their hesitation to take TCM. Up to now, no research data has been published regarding the willingness of asymptomatic COVID-19 patients to accept TCM. Thus, we conducted an online cross-sectional study to record the acceptance and attitude towards TCM by asymptomatic COVID-19 patients and analyzed the predictors influencing the patients’ acceptance of treatment with TCM.

## Materials and Methods

### Study design and participants

An online questionnaire survey was conducted among adult asymptomatic COVID-19 patients who were admitted to the largest fangcang hospital (Si Ye Cao Fangcang hospital) in Shanghai, China, from April 22, 2022, to May 25, 2022. This study employed the “Wenjuanxing” platform to distribute and retrieve electronic questionnaires, and export relevant data information. Patients with intellectual and cognitive impairment were excluded from the survey.

### Ethical approval

The study was approved by the Ethics Committee of The First Affiliated Hospital of Naval Medical University, China (CHEC2022-056). All methods were carried out in accordance with the Declaration of Helsinki. Oral informed consent was obtained and all of the participants were briefed about the purpose of the study, research procedures, privacy of their identity and any other personal information, and their other relevant rights.

### Questionnaires

A self-administered questionnaire was constructed in the Chinese language to evaluate the acceptance and attitude towards TCM. The questionnaire was developed based on the literature review of similar studies and was translated into the Chinese language before distribution to the patients [3, 4, 12–22].

The questionnaire mainly included four parts: (1) Basic information of participants included demographic characteristics (gender, age, body mass index, education level, average monthly income, residence, employment, current smoking status, and current drinking status) and clinical characteristics (comorbidities, vaccination status, and knowledge about culture of TCM). (2) Participants’ attitude toward TCM was determined through the following questions: do you think it takes a long time for TCM to exert efficacy, do you think the TCM treatment is safe, do you think the TCM treatment is effective, will you inform your attending physician if you accept TCM for treatment, and do you think TCM might delay your treatment. (3) Participants’ willingness to take TCM; first, participants were asked to respond to whether they are willing to accept TCM, and if the answer was “Yes”, they were further asked about the types of TCM they were willing to accept and the reasons for their willingness to accept TCM. If the patients were not willing to accept TCM, they were asked the reasons for their unwillingness to accept TCM. We conducted a pilot testing of the main question assessing the willingness to accept TCM among over 50 respondents and did not detect any problems.

### Sample size estimation and data analysis

Based on a previous report [3], the proportion of TCM utilization was assumed as 84% in the calculation of sample size using the following formula:

$$n = Z_{1 - \alpha /2}^2P(1 - P)/{e^2}$$

Where *n* is the minimum number of required patients, Z^2^ indicates 1.96^2^ for 95% confidence interval (CI), *P* presented the estimated utilization rate, and e indicated the required accuracy of 4%. The non-response rate was estimated as 5% and the minimum sample size was calculated as 339 patients.

Data analysis was performed using IBM SPSS Statistics for Windows (version 21.0). Participants’ responses to the questionnaire were treated as classified data, which were expressed by numbers and percentages. Univariate analysis was used to evaluate the relationship between independent variables (participants’ basic information and participants’ attitude towards TCM) and dependent variables (participants’ acceptance towards TCM). The variables with a value of *P* < 0.25 in univariate analysis were put into a multivariate logistic regression analysis to determine the Predictors affecting patients’ willingness to accept TCM. Odds ratio (OR) and 95%CI were adopted to describe these variables. *P* < 0.05 was considered statistically significant.

## Results

### Participants’ characteristics and willingness to accept TCM

A total of 1,185 asymptomatic patients were invited to participate in the survey. One patient submitted an incomplete questionnaire whereas the response was not received from 63 patients. Finally, responses from a total of 1,121 patients were included in the analysis. The overall effective questionnaire recovery rate was 96.60%.

In our study, 1024 participants expressed their willingness to receive TCM, the rate of TCM acceptance was 91.35%. Only 97 participants represented unwillingness to receive TCM, the rate of TCM hesitancy was 8.65% (Table 1).

**Table 1:** Demographic characteristics and clinical features of participants

Item	All participants (*n* = 1121)	Intention to accept TCM		
		TCM hesitancy (*n* = 97)	TCM acceptance (*n* = 1024)	*P*-value
Age (Year)				0.820
< 20	61 (5.44%)	7 (7.22%)	54 (5.27%)	
20–35	511 (45.58%)	41 (42.27%)	470 (45.90%)	
35–50	339 (30.24%)	30 (30.93%)	309 (30.18%)	
> 50	210 (18.73%)	19 (19.59%)	191 (18.65%)	
Gender				0.086
Female	565 (50.40%)	57 (58.76%)	516 (50.39%)	
Male	556 (49.60%)	40 (41.24%)	508 (49.61%)	
BMI (kg/m^2^)				0.198
< 18.5	66 (5.89%)	10 (10.31%)	56 (5.47%)	
18.5–24	634 (56.56%)	49 (50.52%)	585 (57.13%)	
24–28	324 (28.90%)	31 (31.96%)	293 (28.61%)	
> 28	97 (8.65%)	7 (7.22%)	90 (8.79%)	
Residence				0.900
Rural	571 (50.94%)	50 (51.55%)	521 (50.88%)	
Urban	550 (49.06%)	47 (48.45%)	503 (49.12%)	
Education level				0.403
≤ Senior high school	789 (70.38%)	65 (67.01%)	724 (70.70%)	
College degree	175 (15.61%)	14 (14.43%)	161 (15.72%)	
≥ Bachelor’s degree	157 (14.01%)	18 (18.56%)	139 (13.57%)	
Occupation				0.181
Unemployed	257 (22.93%)	26 (26.80%)	231 (22.56%)	
Employed	788 (70.29%)	61 (62.89%)	727 (71.00%)	
Retired	76 (6.98%)	10 (10.31%)	66 (6.45%)	
Average monthly income (CNY)				0.007
< 3000	257 (22.93%)	34 (35.05%)	223 (21.78%)	
3000–8000	635 (56.65%)	42 (43.30%)	593 (57.91%)	
> 8000	229 (20.43%)	21 (21.65%)	208 (20.31%)	
Current smoking status				0.128
No	892 (79.57%)	83 (85.57%)	809 (79.00%)	
Yes	229 (20.43%)	14 (14.43%)	215 (21.00%)	
Current drinking status				0.265
No	1016 (90.63%)	91 (93.81%)	925 (90.33%)	
Yes	105 (9.37%)	6 (6.19%)	99 (9.67%)	
Other underlying diseases				0.242
No	1029 (91.79%)	86 (88.66%)	943 (92.09%)	
Yes	92 (8.21%)	11 (11.34%)	81 (7.91%)	
Have you received two doses of COVID-19 vaccine?				< 0.001
No	112 (9.99%)	20 (20.62%)	92 (8.98%)	
Yes	1009 (90.01%)	77 (79.38%)	932 (91.02%)	
Do you understand the culture of TCM?				< 0.001
No	697 (62.18%)	83 (85.57%)	614 (59.96%)	
Yes	424 (37.82%)	14 (14.43%)	410 (40.04%)	

Data are presented as number (percentage). *P*-values were calculated through univariate analysis between the “TCM hesitancy” and “TCM acceptance” groups. *BMI*: body mass index; *CNY*: China Yuan; *COVID-19*: Coronavirus Disease 2019; *TCM*: traditional Chinese medicine

The relationship of various characteristics with the acceptance of TCM is presented in Table 1. The willingness to accept TCM differed insignificantly (*P* > 0.05) between various demographic characteristics except for the monthly income. Having a monthly income of more than 3,000 yuan, the proportion of participants vaccinated with two doses of COVID-19 vaccines and understanding the culture of TCM were significantly higher for showing a willingness to accept TCM in comparison to those who showed unwillingness.

### Participants’ attitude towards TCM

Table 2 shows significant statistical differences in attitude towards TCM between the TCM hesitancy and TCM acceptance groups (*P* < 0.001 for all the five related questions). In our study, nearly one-fifth of the respondents thought that TCM needed a longer time to take effect. Interestingly, most respondents believed that TCM treatment was safe (1013/1121, 90.37%) and effective (1019/1121, 90.90%), only 11.60% (130/1121) of the participants believed that TCM would delay their treatment.

**Table 2:** Participants’ attitude towards the traditional Chinese medicine

Item	All participants (n = 1121)	Intention to accept TCM		
		TCM hesitancy (n = 97)	TCM acceptance (n = 1024)	*P*-value
Do you think it takes a long time for TCM to exert efficacy?				< 0.001
No	213 (19.00%)	47 (48.45%)	166 (16.21%)	
Yes	908 (81.00%)	50 (51.55%)	858 (83.79%)	
Do you think the TCM treatment is safe?				< 0.001
No	108 (9.63%)	47 (48.45%)	61 (5.96%)	
Yes	1013 (90.37%)	50 (51.55%)	963 (94.04%)	
Do you think the TCM treatment is effective?				< 0.001
No	102 (9.10%)	45 (46.39%)	57 (5.57%)	
Yes	1019 (90.90%)	52 (53.61%)	967 (94.43%)	
Will you inform your attending physician if you accept TCM for treatment?				< 0.001
No	128 (11.42%)	44 (45.36%)	84 (8.20%)	
Yes	993 (88.58%)	53 (54.64%)	940 (91.80%)	
Do you think TCM might delay your treatment?				< 0.001
No	991 (88.40%)	67 (69.07%)	924 (90.23%)	
Yes	130 (11.60%)	30 (30.93%)	100 (9.77%)	

Data are presented as number (percentage). *P*-values were calculated through univariate analysis between the “TCM hesitancy” and “TCM acceptance” groups. *TCM*: traditional Chinese medicine

### Independent predictors of TCM acceptance

According to univariate analysis, 13 variables with *P* < 0.25 were obtained (Tables 1 and 2). Multivariate logistic regression analysis indicated that 6 variables influenced the willingness to accept TCM (*P* < 0.05) (Table 3). The participants who have received two doses of COVID-19 vaccine (OR = 2.069, 95%CI: 1.029–4.162, *P* = 0.041 vs. not received), understood the culture of TCM (OR = 2.293, 95%CI: 1.029–4.162, *P* = 0.014 vs. not understood), thought the TCM treatment is safe (OR = 2.856, 95%CI: 1.334–6.112, *P* = 0.007 vs. not thought), thought the TCM treatment is effective (OR = 2.724, 95%CI: 1.249–5.940, *P* = 0.012 vs. not thought), and those who informed their attending physician if using TCM for treatment (OR = 3.455, 95%CI:1.867–6.392, *P* < 0.001 vs. not informed) were more likely to accept TCM treatment. However, the participants who thought TCM might delay your treatment (OR = 0.256, 95%CI: 0.142–0.462, *P* < 0.001 not thought) was unwilling to accept TCM treatment.

**Table 3:** Predictors of intention to use traditional Chinese medicine

Variable	OR (95% CI)	*P*-value
Gender		
Female	Ref	/
Male	1.787(0.979–3.262)	0.058
BMI (kg/m^2^)		
< 18.5	Ref	/
18.5–24	1.721(0.711–4.167)	0.229
24–28	1.457(0.561–3.782)	0.439
> 28	1.699(0.503–5.740)	0.393
Occupation		
Unemployed	Ref	/
Employed	0.920(0.466–1.814)	0.810
Retired	0.838(0.298–2.355)	0.738
Average monthly income (CNY)		
< 3000	Ref	/
3000–8000	1.254(0.647–2.431)	0.502
> 8000	0.736(0.325–1.666)	0.462
Current smoking status		
No	Ref	/
Yes	1.095(0.512–2.344)	0.815
Other underlying diseases		
No	Ref	/
Yes	0.652(0.270–1.572)	0.341
Have you received two doses of COVID-19 vaccine?		
No	Ref	/
Yes	2.069(1.029–4.162)	0.041
Do you understand the culture of TCM?		
No	Ref	/
Yes	2.293(1.029–4.162)	0.014
Do you think TCM needs a longer time to exert efficacy?		
No	Ref	/
Yes	1.607(0.849–3.034)	0.145
Do you think the TCM treatment is safe?		
No	Ref	/
Yes	2.856(1.334–6.112)	0.007
Do you think the TCM treatment is effective?		
No	Ref	/
Yes	2.724(1.249–5.940)	0.012
Will you inform your attending physician if you accept TCM for treatment?		
No	Ref	/
Yes	3.455(1.867–6.392)	< 0.001
Do you think TCM might delay your treatment?		
No	Ref	/
Yes	0.256(0.142–0.462)	< 0.001

*P*-value indicates whether the adjusted OR of particular sub-category is significant when compared with the reference category. *BMI*: body mass index; *CNY*: China Yuan;*COVID-19*: Coronavirus Disease 2019; *TCM*: traditional Chinese medicine

### The sources of information about TCM

**Fig. 1:**
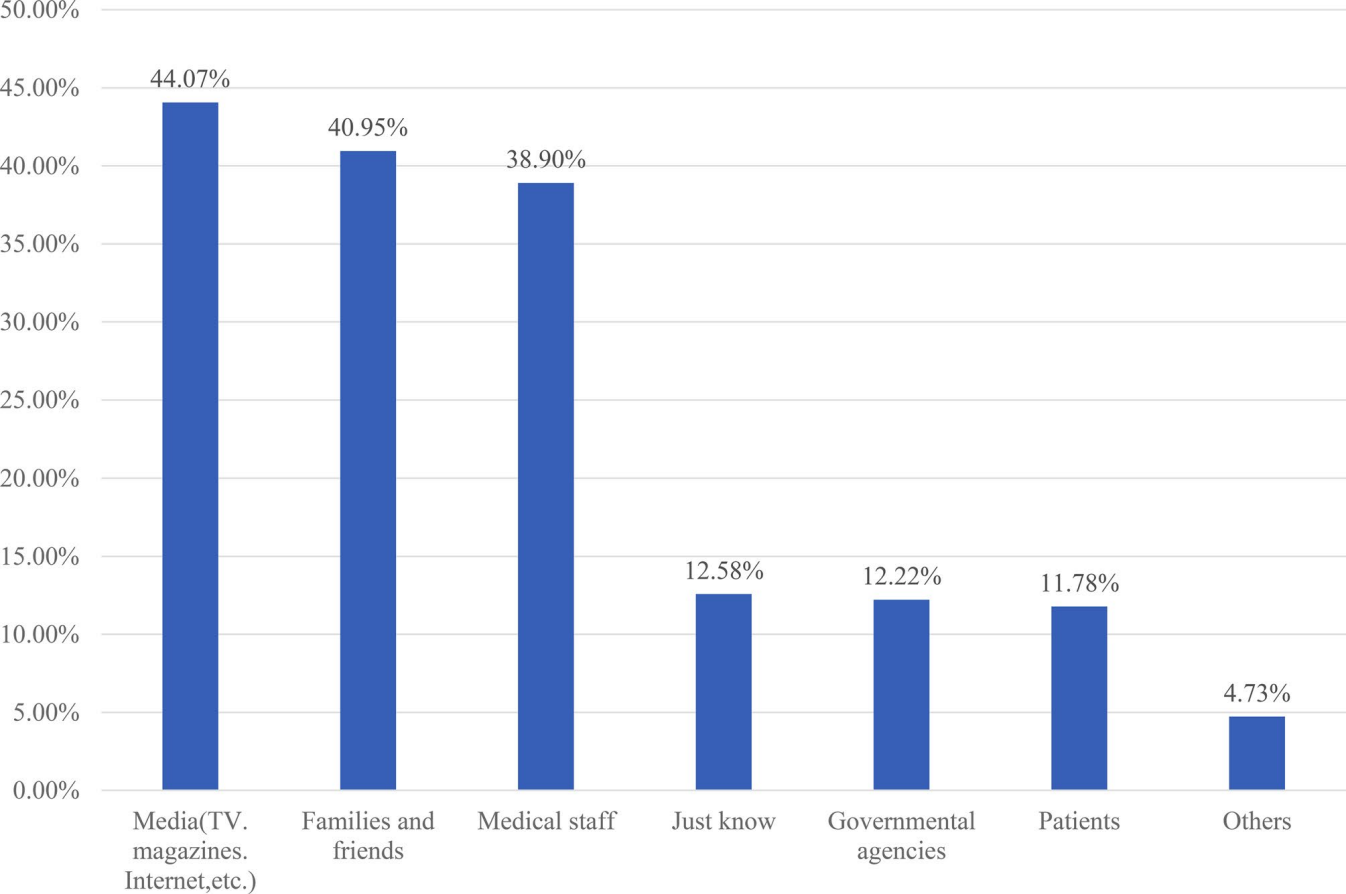
The sources of information about traditional Chinese medicine (n = 1121)

The present study found that the most important source of TCM information for the participants was the media (494/1121, 44.07%), followed by families and friends (459/1121, 40.95%) and medical staff (427/1121, 38.09%) (Fig. 1).

### Reasons for accepting TCM

**Fig. 2:**
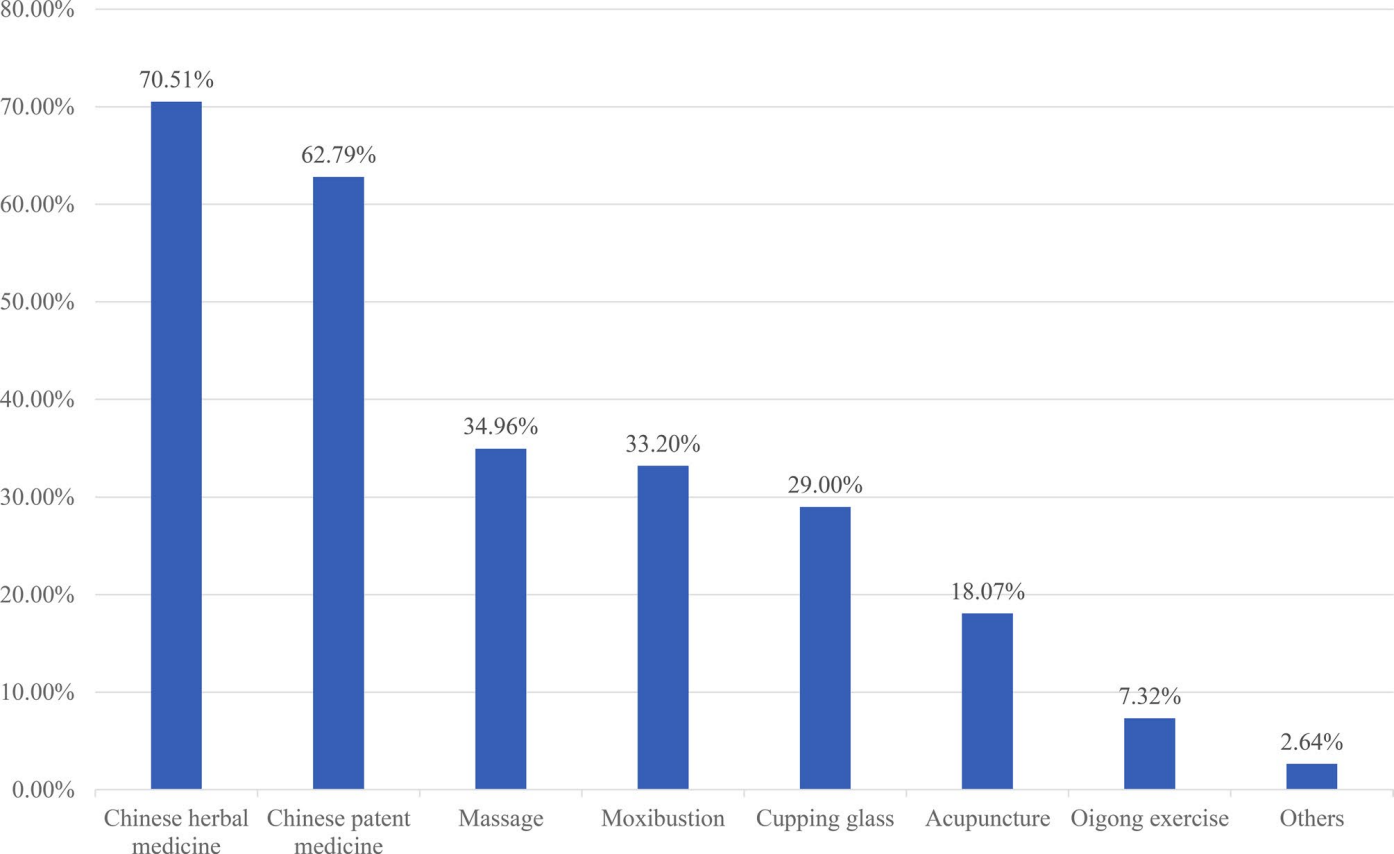
The reasons for willingness to use traditional Chinese medicine among the asymptomatic COVID-19 patients (n = 1024)

Regarding the reasons why 1,024 participants were willing to accept TCM treatment, 51.86% (531/1024) of the participants thought that TCM could improve immunity, and 47.75% (489/1024) believed that TCM could alleviate the symptoms of COVID-19. 44.53% (456/1024) considered that TCM could cure COVID-19 (Fig. 2).

### Types of TCM that the participants were willing to accept

**Fig. 3:**
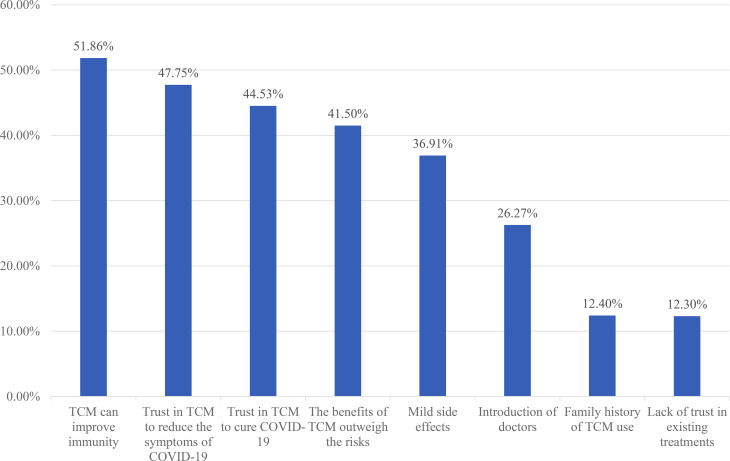
Types of traditional Chinese medicine the asymptomatic COVID-19 patients wanted to accept (n = 1024)

Among the 1,024 participants who were willing to accept TCM, 70.51% (722/1024) were willing to accept Chinese herbal medicine, 62.79% (643/1024) were willing to accept Chinese patent medicine, 34.96% (358/1024) were willing to accept massage, 33.20% (340/1024) chose moxibustion, and 29.00% (297/1024) were willing to accept cupping therapy (Fig. 3).

### Reasons for not accepting TCM

**Fig. 4:**
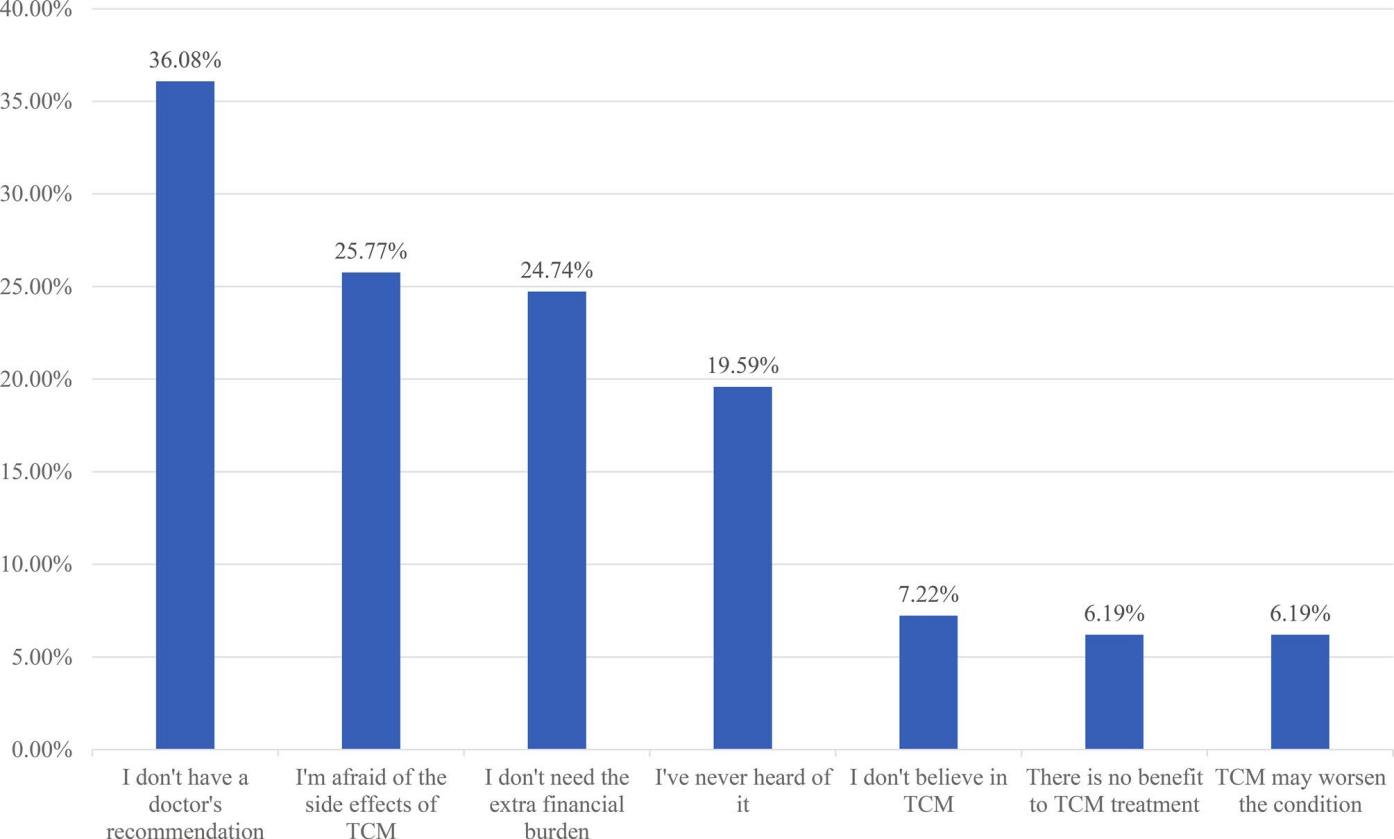
The reasons for reluctance to use traditional Chinese medicine among the asymptomatic COVID-19 patients (n = 97)

For the reasons why 97 respondents were unwilling to accept TCM treatment, 36.08% (35/97) of the respondents reported that they did not receive doctors’ recommendations, and 25.77% (25/97) of the respondents illustrated that they were afraid of the side effects of TCM, and 24.74% (24/97) of respondents showed that they did not need additional burden (Fig. 4).

## Discussion

At present, the COVID-19 pandemic is still spreading all over the world, with constant mutation, which increases the difficulty of prevention and control. TCM has a long history in the prevention and treatment of acute infectious diseases and the experiences of TCM treatment have been recorded in the *Shanghan Lun* and *Detailed Analysis of Epidemic Warm Diseases* [9], which provided a new scheme for treating COVID-19 [23–27]. However, the willingness to accept TCM among asymptomatic COVID-19 patients was not reported in the literature.

The present study explored the acceptance of TCM and its independent predictors among asymptomatic COVID-19 patients and found that 91.35% of the patients were willing to accept TCM treatment, while 8.65% of the patients presented unwillingness to accept TCM. Demographic factors including gender, age, education level, income level, and residence were not significantly associated with TCM acceptance in the present study, which was different from the cross-sectional studies reported from other regions [28–31]. National Health Commission of the People’s Republic of China has repeatedly mentioned the promotion of TCM in the prevention and control of COVID-19, we put forward the following suggestions to accelerate the acceptance of TCM based on the results of this study.

### Increasing the publicity of TCM is important to promote the acceptance of TCM

TCM is a significant part of Chinese cultural heritage, which ideas and practice methods contain the profound wisdom of Chinese philosophy [32]. The multivariate logistic regression analyses indicated that participants who understood the culture of TCM was one of predictors for TCM acceptance, but more than half of the participants lacked an understanding of the culture of TCM in our study. A cross-sectional study in Australia reported that only 26% of dental students knew about CAM [33]. In Hungary, 12.4% of the subjects showed a better understanding of CAM [34]. In Bangladesh, nearly 45% of pharmaceutical students believed that lack of knowledge was the main obstacle to the application of CAM [35]. Given that TCM theory is difficult for non-professionals to understand, a concise and comprehensive introduction of TCM is necessary.

Participants of the present study reported that the most important source of their TCM information was media (44.07%), followed by family members and friends (40.95%) and medical staff (38.09%). In India, the majority of dental practitioners (73.3%) reported that media (internet, newspapers, etc.) was their main source of knowledge about CAM [36]. One study in Silesia, Poland, demonstrated that 60.8% of patients obtained information about CAM from the internet and 38.6% acquired information from television. These results indicated that appropriate media publicity is conducive to guiding and enhancing the public’s receptivity to TCM [37]. This raises requirements for TCM practitioners in China.

### Clarifying the impact of TCM is the premise of advocating TCM

According to the results of the present study, among the participants who were willing to accept TCM, 70.51% preferred Chinese herbal medicine, 62.79% chose Chinese patent medicine, and 34.96% favored massage treatment. A survey on patients with Parkinson disease in China illustrated that herbal medicine, rehabilitation exercise, and acupuncture were the most commonly used TCM therapies [38]. Compared to other TCM therapies, Chinese herbal medicine is the most popular treatment in China.

The safety and efficacy of the TCM is critical to controlling the COVID-19 and has caused widespread concern. Multivariate logistic regression analysis in the current study showed that safety and efficacy of the TCM were the independent predictors for willingness to accept TCM. These results were higher than data from others countries. A survey on diabetic patients in Jeddah Saudi Arabia showed that 54.2% patients believed CAM have no side effect. A study in Indonesia indicated that 68.31% of diabetic patients considered that CAM products were safe and 63.69% of diabetic patients considered that CAM products were effective.

However, considering TCM would delay the treatment was an important independent predictor for hesitation to accept TCM in this study. In fact, the clinical role of TCM in the management of COVID-19 has been verified by clinical trials in China [39, 40]. Therefore, considering the willingness of asymptomatic COVID-19 patients to accept TCM and the effectiveness and safety of the TCM, clarifying the impact of TCM is the premise of advocating TCM for the patients.

### Active communication with attending doctors contribute to acceptance of TCM

Communication between doctors and patients plays an important part in healthcare activities [41], which can determine the patients’ self-management behavior and health outcomes [42]. Our study indicated that informing the attending doctor before receiving TCM treatment is an important predictors for willingness to accept TCM treatment. A study conducted in Palestine from April 2018 to March 2019 exhibited that 64.0% of pregnant women believed that doctors should provide patients with advice on commonly used CAM therapies [43]. In Saudi Arabia, 81.11% of the subjects intended to discuss the application of CAM with their doctors [44]. According to the outpatient service in Iran, among 155 patients who accepted CAM voluntarily, 50 patients (32.2%) reported that they had disclosed the usage of CAM to their doctors [45]. These results suggested that patients had a strong interest in receiving CAM with the advice of their physicians.

In the present study, when participants were asked the reason behind their willingness to accept TCM treatment, only 26.27% of the participants said that it was due to the recommendation by medical staff. When asked about their hesitation toward TCM treatment, 36.08% of participants replied that it had not been recommended by medical staff. Therefore, healthcare practitioners must have a certain understanding of CAM so that they can actively communicate with patients and recommend the most appropriate treatment according to the patients’ conditions. According to a survey in Germany, more than half of the patients expressed their interest in CAM consultation and more than 80% of patients expected their attending doctor to have a certain knowledge of CAM [46]. In India, 57.5% of dental practitioners reported that health professionals should be able to provide patients with advice on commonly used CAM methods [36].

## Limitations

This study has several limitations. First of all, this study is a self-administered questionnaire-based cross-sectional study. Thus, the causality cannot be directly deduced and further longitudinal research is needed to verify the possible causal relationship. Secondly, the questionnaire was developed based on multiple questionnaires but it could not be verified in a large sample size due to time constraints. Thirdly, the sample had a low representation of the elderly (> 60 years old) owing to few admissions of participants of this age to the fangcang hospital. Fourthly, since our survey was just conducted in the largest fangcang hospital in Shanghai, it cannot fully represent the willingness of asymptomatic COVID-19-infected people in Shanghai and even in the world. Fifthly, as the world is urgently fighting against COVID-19, more outcomes of TCM treatment for COVID-19 will be published, which may affect the willingness of patients to accept TCM.

## Conclusion

This study reported the attitude and willingness to accept TCM in asymptomatic COVID-19 patients and their predictors worldwide. In this survey, 91.35% of the participants were willing to accept CAM treatment, while 8.65% of the participants were not willing to accept CAM treatment. Receiving two dose of COVID-19 vaccine, understanding the culture of TCM, thinking TCM is safe and effective, and informing the attending doctor before using TCM were contributors to TCM acceptance, whereas the main contributors to TCM hesitancy was considering that TCM would delay the treatment. Thus, strengthening publicity through the media, especially the emerging network media such as WeChat, microblog, and Zhihu, to generalize the advantages of TCM to the public, inviting the recovering COVID-19 patients who were treated with TCM to give health lectures and broadcasting the clinical practice of TCM in the treatment of COVID-19 in asymptomatic COVID-19 patients, and recommending TCM and teaching some gongfa of TCM (tai chi, qi gong, wuqinxi, etc.) to benefit patients by attending doctors may expand the acceptance of TCM. Further observation will be carried out on this batch of patients to calculate the final prevalence rate of TCM and a long-term follow-up will be conducted to observe the effects of TCM on the quality of life of patients with COVID-19.

## Data Availability

The dataset supporting the conclusions of this article can be made available from the corresponding author on reasonable request.

## Abbreviations

CAM: Complementary and Alternative Medicine
TCM: Traditional Chinese Medicine
COVID-19: Coronavirus Disease 2019

## References

[1] World Health Organization. WHO Coronavirus (COVID-19) Dashboard. (2023-01-27) [2023-01-27]. https://covid19.who.int/.

[2] National Center for Complementary and Integrative Health0. Complementary, alternative, or integrative health: What’s in a name? (2022-05-09) [2022-05-09]. https://www.nccih.nih.gov/health/complementary-alternative-or-integrative-health-whats-in-a-name#hed1.

[3] Dehghan M, Ghanbari A, Ghaedi Heidari F, Mangolian Shahrbabaki P, Zakeri MA (2022). Use of complementary and alternative medicine in general population during COVID-19 outbreak: a survey in Iran. J Integr Med.

[4] Kretchy IA, Boadu JA, Kretchy JP, Agyabeng K, Passah AA, Koduah A (2021). Utilization of complementary and alternative medicine for the prevention of COVID-19 infection in Ghana: a national cross-sectional online survey. Prev Med Rep.

[5] Badakhsh M, Dastras M, Sarchahi Z, Doostkami M, Mir A, Bouya S (2021). Complementary and alternative medicine therapies and COVID-19: a systematic review. Rev Environ Health.

[6] Hong J, Xu XW, Yang J, Zheng J, Dai SM, Zhou J (2022). Knowledge about, attitude and acceptance towards, and predictors of intention to receive the COVID-19 vaccine among cancer patients in Eastern China: a cross-sectional survey. J Integr Med.

[7] Liu J, Manheimer E, Shi Y, Gluud C (2004). Chinese herbal medicine for severe acute respiratory syndrome: a systematic review and meta-analysis. J Altern Complement Med.

[8] National Health Commission of the People’s Republic of China. Notice on issuing the diagnosis and treatment plan for pneumonia infected by novel coronavirus (trial ninth edition). (2022-03-15) [2022-05-09]. http://www.nhc.gov.cn/yzygj/s7653p/202203/b74ade1ba4494583805a3d2e40093d88.shtml.

[9] Zhao C, Li L, Yang W, Lv W, Wang J, Guo J (2021). Chinese Medicine Formula Huashibaidu Granule Early treatment for mild COVID-19 patients: an unblinded, cluster-randomized clinical trial. Front Med (Lausanne).

[10] World Health Organization. WHO Expert Meeting on Evaluation of Traditional Chinese Medicine in the Treatment of COVID-19. (2022-03-15) [2022-05-09]. https://www.who.int/publications/m/item/who-expert-meeting-on-evaluation-of-traditional-chinese-medicine-in-the-treatment-of-covid-19.

[11] Zhang X, Zhang W, Chen S (2022). Shanghai’s life-saving efforts against the current omicron wave of the COVID-19 pandemic. Lancet.

[12] Hwang JH, Cho HJ, Im HB, Jung YS, Choi SJ, Han D (2020). Complementary and alternative medicine use among outpatients during the 2015 MERS outbreak in South Korea: a cross-sectional study. BMC Complement Med Ther.

[13] Pokladnikova J, Park AL, Draessler J, Lukacisinova A, Krcmova I (2021). The use of complementary and alternative medicine by adults with allergies: a czech national representative survey. BMC Complement Med Ther.

[14] Stub T, Jong MC, Kristoffersen AE (2021). The impact of COVID-19 on complementary and alternative medicine providers: a cross-sectional survey in Norway. Adv Integr Med.

[15] Kristoffersen AE, Quandt SA, Stub T (2021). Use of complementary and alternative medicine in Norway: a cross-sectional survey with a modified norwegian version of the international questionnaire to measure use of complementary and alternative medicine (I-CAM-QN). BMC Complement Med Ther.

[16] Sari Y, Anam A, Sumeru A, Sutrisna E (2021). The knowledge, attitude, practice and predictors of complementary and alternative medicine use among type 2 diabetes mellitus patients in Indonesia. J Integr Med.

[17] Ding A, Patel JP, Auyeung V (2020). Testing the traditional Chinese Medicine Consultation Model for Adherence in complementary and alternative medicine. Evid Based Complement Alternat Med.

[18] Owusu S, Gaye YE, Hall S, Junkins A, Sohail M, Franklin S (2020). Factors associated with the use of complementary and alternative therapies among patients with hypertension and type 2 diabetes mellitus in western Jamaica: a cross-sectional study. BMC Complement Med Ther.

[19] Chang HY, Wallis M, Tiralongo E (2012). Predictors of complementary and alternative medicine use by people with type 2 diabetes. J Adv Nurs.

[20] Loquai C, Dechent D, Garzarolli M, Kaatz M, Kaehler KC, Kurschat P (2017). Use of complementary and alternative medicine: a multicenter cross-sectional study in 1089 melanoma patients. Eur J Cancer.

[21] Vlieger AM, van Vliet M, Jong MC (2011). Attitudes toward complementary and alternative medicine: a national survey among paediatricians in the Netherlands. Eur J Pediatr.

[22] Jain L, Vij J, Satapathy P, Chakrapani V, Patro B, Kar SS (2021). Factors influencing COVID-19 vaccination intentions among College students: a cross-sectional study in India. Front Public Health.

[23] Lee DYW, Li QY, Liu J, Efferth T (2021). Traditional chinese herbal medicine at the forefront battle against COVID-19: clinical experience and scientific basis. Phytomedicine.

[24] Shi N, Guo L, Liu B, Bian Y, Chen R, Chen S (2021). Efficacy and safety of chinese herbal medicine versus lopinavir-ritonavir in adult patients with coronavirus disease 2019: a non-randomized controlled trial. Phytomedicine.

[25] Chan KW, Wong VT, Tang SCW (2020). COVID-19: an update on the Epidemiological, Clinical, preventive and therapeutic evidence and guidelines of integrative chinese-western medicine for the management of 2019 Novel Coronavirus Disease. Am J Chin Med.

[26] Lu ZH, Yang CL, Yang GG, Pan WX, Tian LG, Zheng JX (2021). Efficacy of the combination of modern medicine and traditional chinese medicine in pulmonary fibrosis arising as a sequelae in convalescent COVID-19 patients: a randomized multicenter trial. Infect Dis Poverty.

[27] Xiao M, Tian J, Zhou Y, Xu X, Min X, Lv Y (2020). Efficacy of Huoxiang Zhengqi dropping pills and Lianhua Qingwen granules in treatment of COVID-19: a randomized controlled trial. Pharmacol Res.

[28] Abuelgasim KA, Alsharhan Y, Alenzi T, Alhazzani A, Ali YZ, Jazieh AR (2018). The use of complementary and alternative medicine by patients with cancer: a cross-sectional survey in Saudi Arabia. BMC Complement Altern Med.

[29] Ciarlo G, Ahmadi E, Welter S, Hübner J (2021). Factors influencing the usage of complementary and alternative medicine by patients with cancer. Complement Ther Clin Pract.

[30] Ataman H, Aba YA, Güler Y (2019). Complementary and alternative medicine methods used by turkish infertile women and their effect on quality of life. Holist Nurs Pract.

[31] Peltzer K, Pengpid S (2018). Prevalence and determinants of traditional, complementary and alternative Medicine Provider Use among adults from 32 countries. Chin J Integr Med.

[32] Liu C, Gu M (2011). Protecting traditional knowledge of chinese medicine: concepts and proposals. Front Med.

[33] Park JS, Page A, Turner E, Li J, Tennant M, Kruger E (2020). Dental students’ knowledge of and attitudes towards complementary and alternative medicine in Australia - An exploratory study. Complement Ther Med.

[34] Soós S, Jeszenői N, Darvas K, Harsányi L (2016). Complementary and alternative medicine: attitudes, knowledge and use among surgeons and anaesthesiologists in Hungary. BMC Complement Altern Med.

[35] Saha BL, Seam MOR, Islam MM, Das A, Ahamed SK, Karmakar P (2017). General perception and self-practice of complementary and alternative medicine (CAM) among undergraduate pharmacy students of Bangladesh. BMC Complement Altern Med.

[36] Suganya M, Vikneshan M, Swathy U (2017). Usage of complementary and alternative medicine: a survey among indian dental professionals. Complement Ther Clin Pract.

[37] Kasprzycka K, Kurzawa M, Kucharz M, Godawska M, Oleksa M, Stawowy M (2022). Complementary and alternative Medicine Use in Hospitalized Cancer Patients-Study from Silesia, Poland. Int J Environ Res Public Health.

[38] Pan XW, Zhang XG, Chen XC, Lu Q, Hu YS, Han LY (2020). A survey of application of complementary and alternative medicine in chinese patients with Parkinson’s Disease: a pilot study. Chin J Integr Med.

[39] Xia L, Shi Y, Su J, Friedemann T, Tao Z, Lu Y (2021). Shufeng Jiedu, a promising herbal therapy for moderate COVID-19:antiviral and anti-inflammatory properties, pathways of bioactive compounds, and a clinical real-world pragmatic study. Phytomedicine.

[40] Xiong Y, Tian Y, Ma Y, Liu B, Ruan L, Lu C (2022). The effect of Huashibaidu formula on the blood oxygen saturation status of severe COVID-19: a retrospective cohort study. Phytomedicine.

[41] Ong LM, de Haes JC, Hoos AM, Lammes FB (1995). Doctor-patient communication: a review of the literature. Soc Sci Med.

[42] Matusitz J, Spear J (2014). Effective doctor-patient communication: an updated examination. Soc Work Public Health.

[43] Quzmar Y, Istiatieh Z, Nabulsi H, Zyoud SH, Al-Jabi SW (2021). The use of complementary and alternative medicine during pregnancy: a cross-sectional study from Palestine. BMC Complement Med Ther.

[44] Alhawsawi TY, Alghamdi M, Albaradei O, Zaher H, Balubaid W, Alotibi HA (2020). Complementary and alternative medicine use among ischemic stroke survivors in Jeddah, Saudi Arabia. Neurosciences (Riyadh).

[45] Behnood-Rod A, Afzali Poor Khoshkbejari M, Pourzargar P, Hassanzadeh M, Moharamzad Y, Foroughi F (2018). Complementary and alternative medicine use among iranian patients attending urban outpatient general practices. Complement Ther Clin Pract.

[46] Lederer AK, Baginski A, Raab L, Joos S, Valentini J, Klocke C (2021). Complementary medicine in Germany: a multi-centre cross-sectional survey on the usage by and the needs of patients hospitalized in university medical centers. BMC Complement Med Ther.

